# Next-Generation Regenerative Therapies for Alpha-1 Antitrypsin Deficiency: Molecular Pathogenesis to Clinical Translation

**DOI:** 10.3390/ijms26178504

**Published:** 2025-09-01

**Authors:** Se-Ran Yang, Hyung-Ryong Kim

**Affiliations:** 1 Department of Thoracic and Cardiovascular Surgery, School of Medicine, Kangwon National University, Chuncheon 24341, Republic of Korea; 2 Department of Pharmacology, College of Dentistry, Jeonbuk National University, Jeonju 54896, Republic of Korea

**Keywords:** AATD, neutrophil, COPD, RAGE, AEC2, iPSC

## Abstract

Alpha-1 antitrypsin deficiency (AATD) represents a paradigmatic genetic disorder with well-characterized hepatic manifestations but relatively underexplored pulmonary implications. While liver involvement has been extensively reviewed, the underlying mechanisms of lung disease progression remain poorly understood, particularly regarding immunological pathways and inflammatory processes. The pathophysiology involves defective alpha-1 antitrypsin (AAT) production, including AAT variants that induce neutrophil elastase activity, causing progressive alveolar destruction and sustained inflammation, leading to emphysema, as one of the main components of chronic obstructive pulmonary disease (COPD). AATD and smoking represent major risk factors for COPD, the third leading cause of death worldwide at present. In AATD patients, neutrophils, which constitute the majority of circulating leukocytes, become dysregulated. Under normal conditions, cells perform essential functions, including phagocytosis and neutrophil extracellular trap formation (NETosis); in AATD, however, they accumulate excessively in alveolar spaces due to impaired elastase control. The accumulation of Z-AAT polymers within epithelial cells creates a pathological cycle, acting as chemoattractants that sustain pro-inflammatory responses and contribute to chronic obstructive pulmonary disease development. In addition, monocytes, representing a smaller fraction of leukocytes, migrate to inflammatory sites and differentiate into macrophages while secreting AAT with anti-inflammatory properties. However, in PiZZ patients, this protective mechanism fails, as polymer accumulation within cells reduces both AAT secretion and the number of protective human leukocyte antigen(HLA)-DR-monocyte subsets. In particular, macrophages demonstrate remarkable plasticity, switching between pro-inflammatory M1 (classically activated macrophages) and tissue-repairing M2 (alternatively activated macrophages) phenotypes based on environmental cues. In AATD, this adaptive capability becomes compromised due to intracellular polymer accumulation, leading to impaired phagocytic function and dysregulated cytokine production and ultimately perpetuating chronic inflammation and progressive tissue damage. Recent advances in induced pluripotent stem cell (iPSC) technology have facilitated alveolar epithelial cell (AEC) generation, in addition to the correction of AATD mutations through gene editing systems. Despite the limitations of AAT correction, iPSC-derived organoid models harboring AATD mutations can deliver important insights into disease pathophysiology, while gene editing approaches help demonstrate causality between specific mutations and observed phenotypes. Therefore, in this review, we investigated recent studies that can serve as tools for gene editing and drug development based on recently developed iPSC-related technologies to understand the pathogenesis of AATD.

## 1. Introduction

Alpha-1 antitrypsin deficiency (A1AD or AATD) affects approximately 1 in 2000–5000 individuals worldwide and is one of the most common genetic disorders. Alpha-1 antitrypsin (AAT) is a serpin synthesized primarily in the liver and secreted into the circulation. In the liver, point mutations in AAT retention occur, leading to hepatic diseases; in the lung, in comparison, A1AT deficiency induces emphysema. AATD and smoking are major risk factors for the development of chronic obstructive pulmonary disease (COPD), representing the third leading cause of death worldwide [1]. Measurement of A1AT levels is recommended in COPD patients, with AATD now being considered a prevalent genetic form of COPD. Although it stands as one of the genetic disorders, to date, there is no cure for AATD; prompt diagnosis may impede the loss of lung function. In this review, we summarize the current knowledge on the pathophysiology of AATD and describe how organoid-based systems could aid in better understanding AATD and related conditions.

AAT is coded by the serpin family A member 1 (SERPINA1) gene, which is a highly polymorphic 12.2 kb gene clustered with other serpin genes on the long arm of chromosome 14 at position 32.13 (14q32.13), and then synthesized by hepatocytes. Protein folding is required for its release from the endoplasmic reticulum, and it is secreted through the Golgi apparatus [2]. More than 600 SERPINA1 variants have been identified to date, with co-dominant expression of the two alleles. Deficient alleles are associated with Z, M_malton_, and Siiyama, related to protein misfolding and retention in the endoplasmic reticulum. Of note, the Z allele is the most common deficient allele associated with emphysema, reported to be responsible for roughly 95% of clinically diagnosed AATD–emphysema cases. Two alleles comprise a protein inhibitor (PI) genotype (MM), and there is ongoing debate as to how heterozygous genotypes are related to an increased risk of emphysema. PI genotypes and plasma levels of AAT are described herein, and the risk of emphysema is detailed in Table 1. With PI SZ and PI MZ heterozygous genotypes, the risk of lung disease increases depending on whether an AATD patient smokes [3]. Regarding the PI SZ genotype, ever smokers exhibit a higher risk than PI MM or PI MS genotyped smokers. Epidemiologically, it has been demonstrated that the PI MM genotype is most prevalent among individuals with this condition. In the DTC genetic study, of the 195,000 individuals included, 58.1% possessed the PI MM genotype, 28.3% possessed the PI MS genotype, 12.1% possessed the PI MZ genotype, 0.6% possessed the PI SZ genotype, and 0.2% possessed the PI ZZ genotype [4].

AAT is recognized as a protein primarily produced and secreted by the liver, and altered AAT protein production leads to retention within liver tissue. At present, it is widely established that AATD-related liver disease displays a biphasic pattern, initially appearing in infancy and early childhood (0–5 years) with subsequent resurgence during adulthood [5]. Early diagnosis is common in pediatric cases, with most patients experiencing spontaneous resolution of hepatic dysfunction. Progressive liver failure requiring transplantation occurs in only 2–3% of children with the PiZZ phenotype. Although adult AATD patients similarly present with significant hepatic complications, adult PiZZ patients face the additional risk of developing primary liver cancers (PLCs). In American and Canadian patient groups, hepatic steatosis occurred in over 40% of the study cohorts, substantially exceeding the 20–30% rate, indicating that both hepatic steatosis and progressive liver fibrosis are more likely attributable to the AATD-PiZZ genetic variant itself than concurrent metabolic dysfunction-associated steatohepatitis (MASH) pathology [6]. While AAT-mediated pathogenesis and disease mechanisms in hepatic tissues have been established, pulmonary investigations have yet to be as extensively explored. There is thus an urgent need to explore the role of AAT within lung tissue, particularly focusing on the alveoli where gas exchange with the external environment occurs, and investigate the inflammatory and immunological mechanisms involved.

Induced pluripotent stem cell (iPSC) technology have developed therapeutic strategies for AATD and COPD. IPSCs are emerging as particularly valuable models for investigating genetic diseases, offering to determine disease mechanisms in human-derived cellular systems. The development of organoid has further revolutionized this field, addressing the limitations of traditional two-dimensional cell culture models. On the other hand, organoids are able to recapitulate the heterogenous three-dimensional architecture of tissues, providing more physiologically relevant platforms for disease modeling and drug discovery. Research based on patient-derived iPSCs for treating AATD is being conducted, and therapeutic approaches through gene correction are being explored. In this review, we review current understanding regarding AATD pathophysiology and discuss how organoid-based approaches may facilitate into A1AT and associated diseases.

## 2. AATD-Associated Pathway in the Liver and Lung

### 2.1. Liver Injury

AATD represents a complex pathophysiological process initiated by the severe homozygous Pi*Z variant, which causes an 85% decrease in secreted protein and triggers a cascade of cellular dysfunction. The mutant Z-AAT protein accumulates within the endoplasmic reticulum (ER) at the production site, where it creates a proteotoxic environment that fundamentally alters normal cellular homeostasis. The cellular response to the mutant Z-AAT protein misfolding involves two primary degradation pathways employed in an attempt to manage the proteotoxic burden: ER-associated degradation (ERAD) induces monomeric Z-AAT by ER mannosidase I, which removes the protein from the calnexin–calreticulin refolding pathway, whereas autophagy serves as the degradation system for Z-AAT polymerization [7]. In contrast, Z-AAT expression triggers an ER overload response that activates multiple signaling cascades, including nuclear factor kappa B (NF-κB) and mitogen-activated protein kinase (MAPK) pathways. NF-κB activation provides a protective response, promoting protein degradation, while JNK phosphorylation and increased expression of the proapoptotic protein C/EBP homologous protein (CHOP, also known as DNA damage inducible transcript 3 (DDIT3)) accelerate cell death and the development of liver injury via the transcriptional upregulation of SERPINA1. This c-Jun N-terminal kinase (JNK) activation also triggers Forkhead box O3 (FOXO3) activation and upregulates microRNA-34b/c expression, which has complex downstream effects on tissue remodeling and fibrosis development. Furthermore, mitochondrial injury represents an additional consequence of Z-AAT accumulation observed both in Pi*Z mice and in human liver specimens. In Pi*Z mice, overexpression of hepatitis B surface protein was found to exacerbate hepatic damage, fibrotic changes, and hepatocarcinoma incidence; in comparison, the modest iron accumulation seen in homeostatic iron regulator (Hfe) gene knockout mice did not show a significant effect [8].

### 2.2. Lung Injury

AAT is a serine protease inhibitor that belongs to the serpin family. Serpins are involved in various diseases and processes such as angioedema, neurodegenerative diseases, and coagulation [9,10]. During pulmonary circulation, the role of AAT is primarily associated with inactivating certain proteases, particularly neutrophil elastase, in the lungs. Non-impeded neutrophil elastase from activated neutrophils leads to alveolar destruction in the progression of emphysema. The released neutrophil elastase degrades elastin fibers of the alveoli, ultimately resulting in the loss of elastic properties, which is a major feature of emphysema. During sustained neutrophil elastase release, persistent inflammation occurs with neutrophil influx into alveoli through the release of pro-inflammatory cytokines and chemokines. In recent studies, the formation of neutrophil extracellular traps (NETs) has been found to be involved in promoting the activation of lung fibroblasts and myofibroblast transmission. Levels of NETs were found to be increased in the sputum of stable COPD patients and during exacerbations. Upregulation of NET formation in COPD patients was observed alongside higher concentrations of extracellular DNA, and extracellular DNA/NET levels were correlated with those measured based on FEV1 and exacerbation frequency [11]. The increase in NET formation triggers C-X-C motif chemokine ligand-8 (CXCL-8) and TNF-α production, activated platelets, and bacterial products [12]. AAT is a naturally occurring serine protease inhibitor that interacts with neutrophil elastase. Hudock and colleagues demonstrated that AAT limits the NET degradation of E-cadherin to restore barrier function by inducing alveolar epithelial cell death via apoptosis [13]. Cigarette smoking has been proven to decrease lung function in AATD patients by increasing the accumulation of reactive oxygen species, and further increases in reactive oxygen species levels and pro-inflammatory stimulation promote the polymerization of Z-AAT, indicating that plasma deficiency and reduced inhibitory activity of Z-AAT exacerbate emphysema [14]. In addition, the proportion of alveolar macrophages exhibits a marked increase in COPD patients displaying impaired phagocytic activity and increased pro-inflammatory cytokine levels. In one study exploring AATD, excessive misfolded ZAAT of alveolar macrophages induced the unfolded protein via NF-κB pathways, and increased ZAAT polymer levels acted as a potent chemoattractant factor for neutrophils [15].

Alveolar type II epithelial cells (AEC2) are widely recognized as essential in alveolar renewal, repair, homeostasis, and maintenance. In particular, AEC2 are capable of differentiating into alveolar type I epithelial cells (AEC1), which cover 95% of the alveolar surface area and are involved in gas exchange during respiration. Previously described, AATD has been associated with the development of COPD by permanent destruction of the alveolar unit and small airway structures caused by protease expression, apoptosis and an enhanced inflammatory response. Over the past decade, multiple extracellular roles of A1AT have emerged indicating that A1AT prevents caspase activity, nitric oxide production, ER stress, and epithelial barrier disruption. Since Venembre and colleagues reported the ability of AEC2 to produce A1AT, AEC2 have emerged the pivotal cellular target in the pathogenesis of AATD and COPD [16]. A1AT protein has been secreted in alveolar epithelium suggesting that AAT protein expressing AEC2 undergoes intrinsic cellular stress among resident lung cells in ZZ-AATD. AEC2 and alveolar macrophages in ZZ-AATD exhibited a distinct transcriptomic signature including inflammatory pathway and unfolded protein response gene set enrichment [17]. We have reported that the receptor for advanced glycation end product (RAGE) acts as a crucial surface marker on AECs, mediating a variety of inflammatory and cell damage responses, which are central to the pathogenesis of emphysema [18,19]. RAGE is present at a low level in most healthy tissues; however, it is abundant in the lungs, particularly in AECs. Various pathological processes, including those involved in Alzheimer’s disease, atherosclerosis, and cancer, have been associated with highly elevated levels of RAGE [20,21,22]. In our previous study, when analyzing the relationship between the expression of RAGE ligands and COPD patients, COPD patients exhibited higher expression of RAGE binding-related genes such as S100 calcium-binding protein (S100)A2, S100A9, and S100A8. In RAGE-deficient mice, lower inflammatory responses and inhibition of MMP expression were observed, suggesting that RAGE-mediated signaling contributes significantly to oxidative stress and inflammatory responses in the lungs, inducing emphysema [18]. Regarding the AATD-RAGE axis, Ohlmeier et al. reported that the accumulation of the C-terminal-processed variant RAGE increased in the lungs of patients with AAT, a disease in which the emphysematous disease component predominates [23]. Conversely, this finding suggests that loss of RAGE is a marker of tissue damage with fibrotic progress; however, it is not directly associated with pure pulmonary emphysema. AEC2, which produce surfactants, maintain alveoli homeostasis and post-injury repair in the lung and are able to differentiate into AEC1, which facilitates gas exchange. In a recent study, the Tata group suggested that AEC2 progress toward AEC1 differentiation transversely via a distinct pre-alveolar type 1 transitional state prior to terminal maturation. Pre-alveolar type-1 transitional cell state (PAT) undergoes extensive stretching during differentiation, making AEC2 vulnerable to DNA damage via TP53 signaling [24]. Pan and colleagues assessed PATS with CD45^−^Epcam^+^PDPN^high^MHCII^high^ termed “intermediate alveolar epithelial cells” (AECint) and analyzed additional PAT markers, including Sox4, γH2AX, claudin-4 (Cldn4), galectin-3 (LGALS3), and cyclin dependent kinase inhibitor 1A (CDKN1A)/p21, which were found to increase in settings of lung injury caused by bleomycin exposure or human and mouse SARS-CoV-2 infection [25]. Taken together, recent emerging research suggests that PATs, as a potentially vulnerable cellular state during alveolar regeneration, may have significant implications for understanding AATD pathophysiology.

### 2.3. AAT-Associated Pathways in the Inflammatory Response

**Figure 1:**
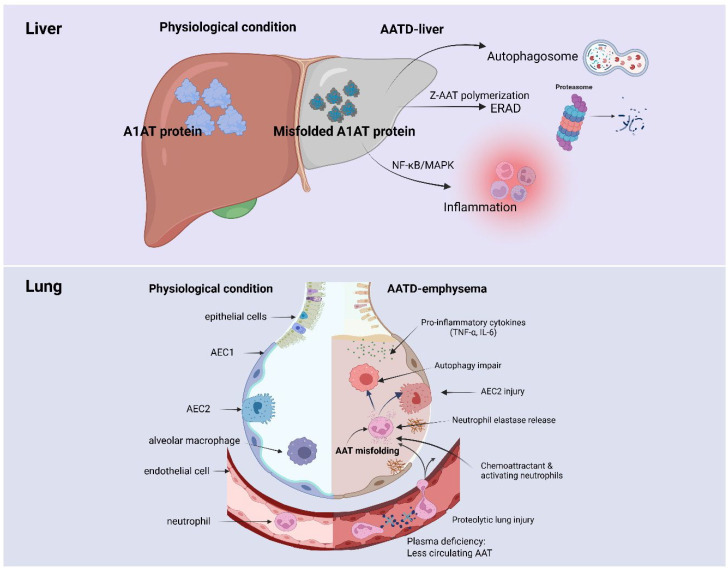
**Pathophysiological mechanism of AATD-associated liver and lung injury**. Under physiological conditions, the liver produces and secretes properly folded alpha-1 antitrypsin (A1AT) protein. In alpha-1 antitrypsin deficiency (AATD), especially due to the Z allele (Z-AAT), misfolded A1AT proteins accumulate in hepatocytes. These misfolded proteins can undergo polymerization and are targeted for degradation via endoplasmic reticulum-associated degradation (ERAD) and autophagy (via autophagosomes and the proteasome). However, the accumulation of misfolded A1AT may trigger activation of inflammatory signaling pathways such as NF-κB and MAPK, leading to hepatic inflammation and liver damage. On the other hand, in bottom panel (Lung): In the lungs, under normal conditions, circulating A1AT inhibits neutrophil elastase, protecting alveolar structures. In AATD-associated emphysema, the lack of functional A1AT results in reduced inhibition of neutrophil elastase. This leads to increased proteolytic lung injury. Additionally, misfolded AAT and plasma deficiency induce epithelial cell (particularly alveolar epithelial type 2 cells, AEC2) injury, impair autophagy, and promote release of pro-inflammatory cytokines such as TNF-α and IL-6. These changes further recruit and activate neutrophils, exacerbating inflammation and lung tissue destruction. Illustration was generated using BioRender.com.

Once AAT is synthesized and released by hepatocytes and immune cells, it circulates in the blood and diffuses. AAT acts through neutrophil elastase (NE), which in turn binds and inhibits proteinases, as well as exhibits anti-inflammatory properties. At an early age, low AAT levels induce the development of early-onset basal panacinar emphysema and injured hepatocytes related to a wide range of liver diseases, the outcomes of which range from portal hypertension and transplant to asymptomatic individuals without fibrosis. Although various mechanisms underlying AATD have been established, its immunological implications remain relatively understudied. The immunological role of AAT represents an underexplored but potentially significant aspect of AATD pathogenesis. Therefore, AAT-mediated immune response may provide insight into potential immunological effects for understanding AATD pathophysiology (Figure 1).

#### 2.3.1. Neutrophils

As neutrophils are generated from hematopoietic stem cells (HSCs) in the bone marrow, they differentiate into mature neutrophils through regulation by granulocyte colony-stimulating factor and chemokines. When inflammation or infection occurs, neutrophil production rapidly increases to enable swift migration to the infection site [26]. In all leukocytes, neutrophils are the most abundant immune cells, comprising 50–70% of leukocytes, and their major functions include phagocytosis, oxidative burst, and neutrophil extracellular trap formation (NETosis). Neutrophils engulf various foreign substances through phagocytosis and effectively eliminate external pathogens. In addition, these cells capture and eliminate pathogens through a specific defense mechanism, NETosis [27]. NETosis is classified into suicidal and vital NETosis depending on NADPH oxidase. This process is induced by external stimuli such as phorbol 12-myristate 13-acetate, lipopolysaccharide, cytokines, cholesterol, and autoantibodies. Phorbol 12-myristate 13-acetate is widely recognized as the most potent NET inducer through activation of MAPK pathways [28]. Lipopolysaccharide binds to the TLR-4 receptor as a ligand through the JNK signaling pathway, inducing NETosis [29]. Taken together, suicidal NETosis is characterized by the release of NET outside the cell as the cell membrane ruptures, and the cells inevitably undergo apoptosis during this process [30,31]. Conversely, vital NETosis independently occurs upon NADPH oxidase activation and progresses rapidly within 30 min. It is induced by external stimuli such as activated platelets, microorganisms, and complement proteins [32]. Once stimulated, calcium ions are imported into neutrophils through small conductance potassium channel member 3, which activates PAD4, leading to citrullination of histone H3. This process weakens the electrostatic binding between histone and DNA, causing chromatin decondensation, and decondensed chromatin is released outside neutrophils via vesicles together with histones and granule proteins [33]. Accordingly, a major difference from suicidal NETosis is that neutrophils survive during this process, and the cell membrane is maintained. NET formation in COPD was first reported in 2015. NETs form mucus rich in large amounts of DNA and proteins, increasing viscosity and consequently obstructing airways, leading to impaired lung function. The patterns of NETs found in the sputum of COPD patients differ according to disease stage. The majority of exacerbated COPD patients exhibit NETs; in comparison, they are found in only approximately 45% of patients with stable COPD, indicating that NETs perpetuate and exacerbate inflammation through various mechanisms [34].

Neutrophils are the primary source of serine proteinases that cause injury to the airways. AAT is secreted as a single polypeptide chain and undergoes an inhibitory mechanism through the oxidation of either methionine 351 or methionine 358 in AATD, leading to loss of anti-neutrophil elastase activity [35]. Patients with AATD exhibit markedly elevated neutrophil accumulation in their alveoli compared with healthy individuals, indicating that increased neutrophil count enhances chemotactic activity. In chemotactic activators, leukotriene B4 (LTB4) represents a key chemotactic stimulus. Defective elastase containment within airways triggers increased alveolar macrophage release of the neutrophil chemoattractant LTB4. In AATD patients, LTB4 is predominantly found to be responsible for neutrophil migration. Of note, neutrophil chemotactic activity has been found to be related to homozygous and heterozygous individuals with the Z allele of AATD; individuals with this allele exhibit greater neutrophil influx into the airways. Multiple mechanistic models have been proposed in an effort to understand the mechanism responsible for increases in neutrophil accumulation in the lungs of AATD patients [36]. Some researchers have proposed that Z-AAT polymers accumulate within both alveolar and bronchial epithelial cells, serving as potent chemoattractants. In the liver, individuals homozygous for ZZ or PIZZ are classified as having AATD, and molecules of Z-AAT polymers are recognized as an unusual conformation of polymers within hepatocytes that are hepatotoxic, leading to liver injury. In children with AATD, high levels of circulating Z-AAT polymers and gamma glutamyl transferase are related to clinically evident portal hypertension [37]. In a recent study, Jagger et al. employed nuclear magnetic resonance (NMR) spectroscopy to examine AATD patients and observed chemically shifted perturbations with the E342K Z-AAT variant in order to examine solution structure and dynamics [38]. Z-AAT polymers promote sustained pro-inflammatory activity and are potentially correlated with the development of COPD through small airway disease impairment; moreover, chronic bronchiolitis is related to increased Z-AAT polymer levels in individuals with Z-related AATD [39]. In addition, it has been demonstrated that neutrophil elastase upregulates TLR4-mediated IL-8 expression of bronchial epithelial cells, leading to the promotion of neutrophil migration and exacerbation of alveolar inflammation [40,41]. In human neutrophils, glycosylated AAT binds to IL-8, and the AAT-IL8 complex prevents IL-8 interaction with CXCR1. AAT modulates neutrophil chemotaxis in response to the soluble immune complex through the glycosylphosphatidylinositol-anchored Fc receptor FcγRIIIb membrane by inhibiting ADAM-17 activity. Shedding of FcγRIIIb derived from the neutrophil membrane is involved in immune complex-stimulated neutrophil accumulation, suggesting that neutrophil chemotaxis requires surface shedding activity such as a disintegrin and metalloprotease domain 17 (ADAM-17) in neutrophils [42,43].

**Figure 2:**
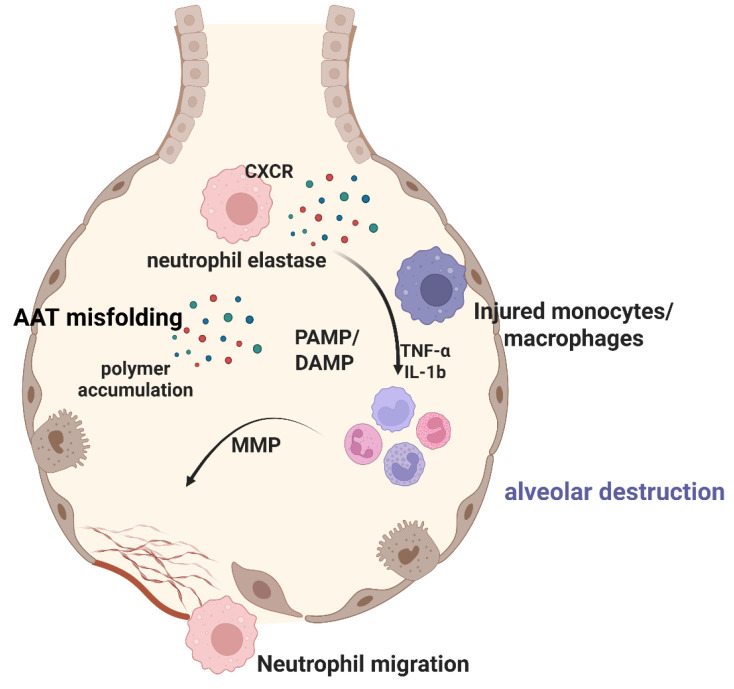
**Pathophysiology of AATD-associated alveolar injury**. This schematic depicts the molecular mechanisms underlying lung damage in alpha-1 antitrypsin (AAT) deficiency. Misfolded AAT proteins accumulate as polymers in lung epithelial cells, reducing functional AAT availability. In response to pathogen-associated molecular patterns (PAMPs) and damage-associated molecular patterns (DAMPs), the neutrophils migrate from the circulation into the alveolar space via CXCR-mediated chemotaxis. These activated neutrophils release elastase, which, in the absence of sufficient functional AAT, remains unopposed and degrades alveolar structural proteins. Injured monocytes and macrophages produce pro-inflammatory cytokines (TNF-α, IL-1β) that perpetuate the inflammatory cascade. Matrix metalloproteinases (MMPs) are activated, further contributing to extracellular matrix degradation. This sustained inflammatory response ultimately leads to progressive alveolar destruction and emphysema. The figure illustrates the key cellular and molecular components involved in the pathological process, highlighting how AATD leads a protease-antiprotease imbalance that drives lung tissue destruction.

The substantial neutrophil accumulation observed in AATD lungs induces proteolytic activity and inflammatory responses. It has been demonstrated that the number of phagocytic cells denotes reduced lung function and elevated concentrations of pro-inflammatory mediators such as IL-8, IL-6, and IL-1β [36]. Matrix metalloproteinase-9 (MMP-9) is the main proteinase related to COPD, and among various biomarkers, MMP-9 expression is highly correlated with COPD exacerbations [42]. Indeed, in one study, the MMP-9 levels of individuals with COPD were found to be higher than those of healthy individuals, and MMP-9 and C-reactive protein were found to be significantly associated with declines in forced expiratory volume in 1 s (FEV1) [44]. The above findings indicate that AATD neutrophils are dysfunctional at the site of misfolded Z-AAT protein accumulation, associated with cellular stress and accelerated neutrophil apoptosis (Figure 2).

#### 2.3.2. Monocytes and Macrophages

Human monocytes comprise approximately 5–10% of circulating blood leukocytes. Monocytes possess specialized chemokines and adhesion receptors that facilitate their migration to the site of injury. When cells migrate, activated monocytes secrete inflammatory cytokines, including IL-1β, IL-6, and TNF-α, together with additional inflammatory substances, which serve to attract additional immune cell types and moderate the inflammatory response. Upon migrating to peripheral tissues, monocytes differentiate into macrophages or dendritic cells based on the surrounding microenvironment. This transformation enhances the tissue’s ability to encapsulate pathogens or present antigens as required, thereby facilitating pathogen elimination and immune responses. During this process, monocytes serve as an essential component in the adaptive immune defense against infection.

Phagocytosis initiates when pattern recognition receptors of phagocytes recognize pathogen-associated molecular patterns (PAMPs) or damage-associated molecular patterns (DAMPs). The innate immune system, unlike the adaptive immune system, is capable of recognizing a broad range of pathogens because innate immune receptors recognize common molecular features shared by pathogens [45]. The receptors of the cells responsible for innate immunity are called pattern recognition receptors (PRRs), and pathogen molecules are recognized by PAMPs. Pathogens often exert specific cellular functions during the process of infection initiation, which is detected by PRRs. In addition, specific molecules expressed by DAMPs, which are recognized by PRRs, may differ in that they originate from the host’s own cells [46]. This PRR-mediated recognition is less specific compared to the antigen–antibody interactions of adaptive immunity; however, it enables rapid innate immune responses. Once pathogens are recognized, neutrophil cellular membranes surround and engulf them to form a phagosome. Subsequently, the phagosome fuses with one or more lysosomes to become a phagolysosome. The interior of the phagolysosome becomes acidified, activating lysosomal hydrolytic enzymes and generating reactive oxygen species (ROS) and reactive nitrogen species (RNS) to destroy pathogens through oxidative reactions [47]. Of note, neutrophils exhibit efficient intracellular pathogen elimination capabilities due to their specialized granules.

During monocyte differentiation into macrophages, AAT is released and retains its secretory capacity. In monocytes, AAT plays a key role in neutralizing free neutrophil elastase, suggesting that AAT secretion exhibits an additional anti-inflammatory effect. In one study, AAT inhibited the lipopolysaccharide-induced release of TNF-α and IL-1β in monocytes while concurrently increasing the level of IL-10 in monocytes [48]. The results of preliminary studies suggest that patients with the PiZZ genetic form of AATD may exhibit lower levels of HLA-DR^-^ monocytes, which are responsible for protecting endothelial cells through the removal of cellular debris and the release of anti-inflammatory mediators [49]. This finding suggests that PiZZ is one of the major factors involved in inducing early inflammation onset; however, this remains an emerging hypothesis requiring further validation in future studies. When exposed to neutrophil elastase, monocytes upregulate AAT mRNA levels; however, PiZZ monocytes release less AAT due to AAT polymer accumulation. In addition, CXCL-8 and IL-6 promote additional neutrophil recruitment to the lung, thereby increasing the elastase load and additionally leading to alveolar destruction in the lungs of AATD patients [50]. Alveolar macrophages maintain extended longevity while being replenished through monocyte recruitment in acute inflammation, generating monocyte-derived macrophages. These macrophages demonstrate phenotypic flexibility and possess the ability to modulate their characteristics in different microenvironments. Both resident macrophages within tissues and inflammatory macrophages undergo activation to become either classically activated macrophages (M1) or alternatively activated (M2) macrophages. M1 macrophages are stimulated by cytokines such as interferon and exert pro-inflammatory functions. M2 macrophages are stimulated by interleukin-4 and interleukin-13, facilitating tissue restoration and inflammatory resolution through the production of anti-inflammatory factor IL-10 [51]. Despite macrophage polarization toward M1 or M2 phenotypes, it remains unclear how alveolar macrophage polarization contributes to molecular mechanisms in AATD patients. Specifically, the excretion of exogenous AAT has been shown to restore efferocytosis and phagocytosis by macrophages exposed to oxidative stress due to decreased CD206 expression and the phosphatidylserine receptor. In one study, PiZZ patients exhibited lower release than healthy subjects, leading to the accumulation of AAT polymers associated with reduced AAT production [52]. Although the exact mechanism of AATD remains unknown, there is an urgent need to comprehend the accumulation of polymers at the cellular level, such as phagocytosis and cytokine release.

## 3. Future Developments

###  Organoid-Based Therapeutic Approach for AATD

Several cellular models of the AATD liver have been developed. In previous studies, patient iPSCs have been differentiated into hepatocyte-like cells, thereby reiterating interindividual variability in the expression of liver disease. It has been demonstrated that, in Pi*ZZ-derived cellular systems, unfolded protein response activation and inflammatory network stimulation are the predominant molecular changes observed. While constrained by limited access to human tissue specimens, the use of hepatic organoids from Lgr5^+^ progenitor cells represent another relevant experimental strategy. In previous studies, Pi*ZZ organoids have been shown to play a role in reducing liver-specific gene synthesis, such as hepatocyte nuclear factor 4 alpha (HNF4α). When hepatic organoids were treated with oncostatin M, a widely recognized inducer of SERPINA1, increased expression of full-length transcripts was noted, in addition to the short transcript of AAT [53]. Although various cell line systems have been developed to express elevated levels of PI*Z or alternative SERPINA mutations, such systems are inherently limited to understanding biological processes specific to fully mature hepatocytes. In this regard, genome-editing systems, including zinc finger nucleases (ZFNs), transcription activator-like effector nucleases (TALENs), and clustered regularly interspaced short palindromic repeats (CRISPRs), have been developed to facilitate desired genomic modifications. The use of these systems in stem cells is indispensable for monogenic defect diseases, considering the fact that stem cells reflect human developmental phases. These benefits have led to various iPSCs undergoing genetic correction through the use of established methods. From this perspective, patient-specific iPSCs hold significant promise for use in the treatment of a diverse range of hereditary diseases, in response to demand for a safe and precise technique for permanent genetic alteration. AATD is caused by defective production of AAT in the liver, and decreased circulating A1AT could causes serious liver damage owing to dysregulated serine proteases [54]. In AATD patients, AAT proteins with the E342K Z-AAT mutation exhibited excessive accumulation, and most mutants formed polymers correlated with alterations in protein structure in hepatocytes. In Kupffer cells (KC)-mediated hepatic inflammation, notably IL-1β led to the transcriptional inhibition of A1AT by HNF4α. Moreover, Serpina1a-e knockout mice, ablation of A1AT worsened metabolic dysfunction-associated steatohepatitis through increased activity of proteinase 3 and F4/80^hi^/CD11b^low^/TIM4^−^/CCR2^+^ monocyte-derived KCs [55]. AAT knockdown with a lentiviral vector microRNA30-style shRNA has been demonstrated in murine iPSCs, with persistent knockdown of high levels of A1AT in the liver of the mouse model. In AATD-patient-derived iPSCs, the use of ZFN and piggyBac technology facilitated the correction of a point mutation (Glu342Lys) in A1AT [56]. As a result, A1AT-corrected iPSCs restored the structure and function of A1AT, resulting in maintenance of the ability to differentiate into cells expressing three germ layers. When the corrected cells were differentiated into hepatocytes, they exhibited significant LDL cholesterol uptake, albumin secretion, and Cytochrome P450 activity [56]. In one study involving AATD patient-derived iPSCs, TALEN was employed, and the corrected cells were differentiated into hepatocytes without mutant AAT accumulation [57]. Choi et al. demonstrated that TALEN in human iPSCs can facilitate higher efficiency than that observed with ZFNs because of similar targeting efficiency, making them an effective, robust, and economic alternative system. Although the established corrected AAT-iPSCs were reliable and free from residual ectopic sequences, 29 mutations in 22 coding exons were still present, with nonsynonymous or splicing mutations. Nevertheless, these findings require confirmation in animal models or clinical specimens. These findings demonstrate that the mutation load may impact the normal germ-line load, and accumulated somatic variation requiring primary screening is critical for safe clinical application. To facilitate the establishment of cellular and animal models for AATD, substantial limitations persist in converting these mechanistic data into clinical analysis. The overwhelming majority of AATD patients, including those with PiZZ variants, remain unidentified and are frequently misdiagnosed with alcohol-associated liver diseases or cryptogenic cirrhosis. Comprehensive genetic analysis for patient screening and identification protocols, in particular, environmental triggers, is therefore vital.

The human lung is composed of more than 50 identified cell types, and they are distributed across various parts of the lung, including the airways and parenchyma [58]. Within the lung, the alveoli are the most distal structures of the respiratory tract, and AEC2 are the main cellular source that can be used to assess alveolar function in gas exchange and lung repair after injury. In certain cells, such as AEC2, not only is the limited cell quantity an issue that poses constraints to their application, but also maintaining their differentiation state, thus necessitating large-scale production of the required cells with proper differentiation for cellular therapeutics. In this regard, human embryonic stem cells (ESCs) and induced pluripotent stem cells (iPSCs) have the developmental potential to differentiate into the expected mature cell types and serve as pivotal tools for studying human diseases and developmental biology [59]. A few research groups have reported iPSC-derived AEC2 protocols that result in lung progenitors expressing alveolar marker genes such as NK2 Homeobox 1 (NKX2-1) and surfactant protein C (SFTPC) [60,61]. Jacob et al. generated dual-targeted iPSCs with NKX2-1^GFP+^/SFTPC^tdTomato+^ reporters and found that SFTPC-expressing cells from NKX2-1 precursors promoted distal airway patterning and alveologenesis [62], highlighting differentiated AEC2 as a reliable surrogate for investigating alveolar disease such as cystic fibrosis. Brownfield et al. demonstrated that fibroblast growth factor receptor 2 (Fgfr2) signaling directly and autonomously restricts AEC2 differentiation to change the fate of AEC1 [63]. This finding suggests that the inhibition of Fgfr2 results in the disruption of alveoli and gas exchange, leading to activation of AEC2 apoptosis. In addition, AEC2 express ACE2 and transmembrane protease serine subtype 2 (TMPRSS2) transcripts encoding genes for SARS-CoV-2 cell entry [64]. Furthermore, alveolar macrophages are emerging as an abundant immune cell population that protects the lung against potentially harmful inhaled agents and maintains optimal lung function by clearing excess or aged surfactant [65]. Indeed, Kang and colleagues developed a coculture system combining iPSC-derived alveolar epithelial organoids and induced macrophages. The assembloids with alveolar epithelial cells and macrophages exhibited efficient elimination of injured cells and absorbed oxidized lipids [66]. Although the study authors agree on the unmet need to target alveolar epithelial cell-related airway distal diseases, identifying the underlying limitations of AEC2 generation remains a time-consuming process that involves an unknown, immature cellular population. Moreover, in the lung, as in other organs, the presence of immune cells such as macrophages represents an important cellular component, yet challenges remain regarding how to optimize culture conditions to preserve the function and properties of these cell sources. Given this challenge, it is critical to develop shorter and more rapid iPSC-derived protocols capable of expanding cells on a large-scale using bioreactors.

Taken together, we have discussed the effects of AAT variants on lung and liver injury and the underlying pathological mechanisms. At present, there are no specific therapeutic interventions available for AATD. While gene correction approaches represent one critical therapeutic avenue for AATD, this review aims to highlight the diverse therapeutic strategies being pursued beyond gene correction. Patient-derived iPSC techniques emerge as a critical approach for clinical application and an individualized treatment approach in the complicated proteotoxic condition. These models provide valuable insights into disease mechanisms across different cell types and organ systems.

## Figures and Tables

**Table 1:** SERPINA1 variants and risk of emphysema.

Genotype	Plasma Level (mg/dL)	Risk of Emphysema
***MM***	100–200	Usual
***MS***	100–180	Usual
***MZ***	66–120	Mild increased
***SS***	70–105	Usual
***SZ***	45–80	Mild increased (20–50%)
***ZZ***	10–40	Markedly increased (80–100%)
***Null***	0	Markedly increased (100%)
